# Deep Sequencing of Chicken microRNAs

**DOI:** 10.1186/1471-2164-9-185

**Published:** 2008-04-22

**Authors:** Joan Burnside, Ming Ouyang, Amy Anderson, Erin Bernberg, Cheng Lu, Blake C Meyers, Pamela J Green, Milos Markis, Grace Isaacs, Emily Huang, Robin W Morgan

**Affiliations:** 1 Department of Animal and Food Sciences, Delaware Biotechnology Institute, University of Delaware, Newark, Delaware, 19711, USA; 2 Department of Plant and Soil Sciences, Delaware Biotechnology Institute, University of Delaware, Newark, Delaware, 19711, USA; 3 University of Louisville, Department of Computer Engineering & Computer Science Louisville, Kentucky, 40292, USA

## Abstract

**Background:**

The use of new, deep sequencing technologies has greatly accelerated microRNA discovery. We have applied this approach to the identification of chicken microRNAs and to the comparison of microRNAs in chicken embryo fibroblasts (CEF) infected with Marek's disease virus (MDV) to those present in uninfected CEF.

**Results:**

We obtained 125,463 high quality reads that showed an exact match to the chicken genome. The majority of the reads corresponded to previously annotated chicken microRNAs; however, the sequences of many potential novel microsRNAs were obtained. A comparison of the reads obtained in MDV-infected and uninfected CEF indicates that infection does not significantly perturb the expression profile of microRNAs. Frequently sequenced microRNAs include miR-221/222, which are thought to play a role in growth and proliferation. A number of microRNAs (e.g., let-7, miR-199a-1, 26a) are expressed at lower levels in MDV-induced tumors, highlighting the potential importance of this class of molecules in tumorigenesis.

**Conclusion:**

Deep sequencing technology is highly suited for small RNA discovery. This approach is independent of comparative sequence analysis, which has been the primary method used to identify chicken microRNAs. Our results have confirmed the expression of many microRNAs identified by sequence similarity and identified a pool of candidate novel microRNAs.

## Background

MicroRNAs are small (about 22 nt) RNAs that play important regulatory roles by targeting mRNAs for degradation or translational repression. MicroRNAs were first identified in *Caenorhabditis elegans *[1] but high evolutionary conservation eventually led to the identification of microRNAs in other species. This, coupled with conventional sequencing of small RNA libraries, has greatly expanded the list of known microRNAs. The most recent release of the microRNA database, miRBase 10.0 [2], contains over 5000 microRNA gene loci in a wide variety of animal, plant and viral genomes.

Conventional sequencing favors identification of highly expressed species, and comparative genomics will not identify nonconserved microRNAs. In order to enhance discovery of small RNA species, massively parallel signature sequencing (MPSS) was used to identify small RNAs in *Arabidopsis thaliana *[3], and the results showed that the diversity of small RNAs exceeded previous estimates. More recently, newer deep sequencing technologies have been used to profile microRNAs in *Arabidopsis *DICER and RDR2 mutants [4,5], and others have applied this technology to various samples including human and chimpanzee brain [6] and *Chlamydomonas reinhardtii *[7]. These approaches have the advantage that they not only provide sequence of low abundance species, but also provide quantitative data since the frequency of sequencing reads reflects the abundance of microRNAs in the population.

We previously reported on the use of deep sequencing technologies for identification of microRNAs encoded by Marek's disease virus (MDV), an economically important pathogenic herpesvirus of chickens [8,9]. In an extension of the pilot study, we sequenced additional reads from both MDV-infected chicken embryo fibroblasts (CEF) and uninfected CEF and now report on the identification of potential novel host microRNAs. In addition, the sequence of several new MDV-encoded microRNAs were discovered by deeper sequencing.

## Results

### Small RNA libraries

We obtained 256,221 reads from two small RNA libraries prepared from uninfected CEF or CEF infected with MDV. As shown in Table 1, a total of 171,783 reads contained both adapters used in creating the library, and 125,463 of these high quality reads showed an exact match to the chicken genome. A total of 1,036 reads from the MDV-infected CEF library matched the MDV genome. The presence of other small RNAs (ribosomal fragments, tRNA, snRNA, mtRNA) was relatively small (less than 3%).

**Table 1:** Distribution of small RNAs from uninfected CEF and CEF infected with MDV

	**MDV infected CEF**	**uninfected CEF**
High quality/both adapters	107,728	64,055
Exact match to chicken genome	79,074	46,389
Match to known miRNAs	67,982	40,173
Match to other chicken smalls^1^	3,249	1,487
Match to MDV	1036	-
Other potential smalls	7,761	4,666

^1^tRNA, rRNA, mtRNA, snRNA

**Figure 1:**
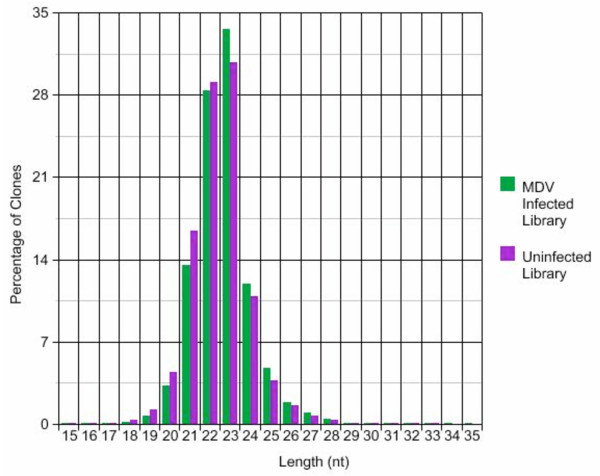
Size distribution of small RNAs.

The majority (86%) of the small RNAs match to known or predicted chicken microRNAs (Additional File 1). Of the 149 distinct *Gallus gallus *(gga) entries in miRbase, we found 101 distinct species expressed in CEF. There were 93 matches from the MDV-infected CEF library and 87 matches from the uninfected CEF library. The infected cells showed slightly more complexity in microRNA diversity, which may be in part due to the larger number of reads obtained from the infected CEF library which increases the chances of revealing low abundance microRNAs. There were 12 microRNAs in the infected cells that were not found in the uninfected CEFs and 9 microRNAs found in the uninfected CEFs that were not found in the infected cells. An additional eleven chicken homologs of known microRNAs were identified (Additional File 1). The size distribution of reads was not significantly different in the two libraries, and the majority of the reads had lengths of 21–25 nt (Figure 1).

### microRNA profiling by analysis of read counts

The number of reads obtained should reflect the relative abundance and expression levels of the microRNAs. After scaling for total number of reads obtained for each library, the majority of microRNAs were found at similar levels in the two libraries. A few microRNAs (listed in Table 2) showed a greater than two-fold difference in the number of reads between the infected and uninfected CEF libraries. We found miR-29b and miR-196 at higher levels in the MDV-infected cells, and three of the let7 microRNAs were found at lower levels in the MDV-infected CEF compared to the uninfected CEF. Northern blot analysis did not detect these differences, but this could be because of the low read numbers or because of cross hybridization with microRNAs with similar sequences (miR-29a, let7 family).

**Table 2:** Relative abundance of differentially expressed microRNAs

**microRNA name**	**Length**	**Sequence**	**# Reads in MDV infected CEF**	**# Reads in uninfected CEF**	**Ratio Infected/Uninfected (Normalized)**
gga-miR-29b	23	TAGCACCATTTGAAATCAGTGTT	64	2	18.8
gga-miR-196	21	TAGGTAGTTTCATGTTGTTGG	23	1	13.5
gga-miR-133a	22	TTGGTCCCCTTCAACCAGCTGT	15	2	4.4
gga-miR-10b	22	TACCCTGTAGAACCGAATTTGT	70	15	2.7
gga-miR-30d	22	TGTAAACATCCCCGACTGGAAG	23	6	2.3
					
gga-let-7f	22	TGAGGTAGTAGATTGTATAGTT	338	454	0.4
gga-let-7b	22	TGAGGTAGTAGGTTGTGTGGTT	74	105	0.4
gga-miR-130a	22	CAGTGCAATATTAAAAGGGCAT	18	28	0.4
gga-let-7a	22	TGAGGTAGTAGGTTGTATAGTT	383	603	0.4
gga-miR-1a	21	TGGAATGTAAAGAAGTATGTA	7	12	0.3

Data shown are microRNAs with more than 15 reads in one library and 2-fold difference in read count, after scaling for total number of reads matching the chicken genome in each library.

The most frequently sequenced (> 500 reads) microRNAs are found at remarkably similar levels in the two libraries (Table 3). Consistent with findings in our pilot study [9], the highest number of reads was obtained for gga-miR-222 and 221. These are clustered on chromosome 1 (114216027–114219024), and in the chicken there are two copies of miR-222 in the cluster, which could account for the higher number of miR-222 reads. We also see high levels of gga-miR-125b/148a/21 and 103.

**Table 3:** Most frequently sequenced microRNAs in CEFs

**microRNA name**	**# Reads in MDV infected CEF**	**# Reads in uninfected CEF**	**Ratio Infected/Uninfected (normalized)**
gga-miR-222a	10361	4945	1.2
gga-miR-221	6708	4112	1.0
gga-miR-125b	3689	2093	1.0
gga-miR-148a	3583	2062	1.0
gga-miR-21	2929	1488	1.2
gga-miR-103	1297	841	0.9
gga-miR-17-5p	826	564	0.9
gga-miR-20a	666	414	0.9
gga-miR-27b	650	400	1.0
gga-miR-20b	619	378	1.0
gga-miR-199	529	301	1.0
gga-miR-26a	522	334	0.9
gga-miR-218	499	370	0.8

Data shown are microRNAs with more than 15 reads in one library and 2-fold difference in read count, after scaling for total number of reads matching the chicken genome in each library.

### Viral microRNAs

We previously identified ten MDV-encoded microRNAs in a pilot sequencing project of the MDV-infected CEF library [9]. The deep sequencing revealed an additional seven microRNAs and '*' strands (Table 4). Four of the new microRNAs map to the previously identified LAT cluster (mdv1-miR-6*, 8*, 10, and 10*), two are in the cluster upstream of the *meq *gene (mdv1-miR-11 and 5*), one is downstream of the *meq *gene (mdv1-miR-12), and one is antisense to the coding region of the *ribonucleotide reductase *gene (mdv1-miR-M9). (A preliminary discussion of some of these microRNAs was reviewed in [8]).

**Table 4:** MDV encoded microRNAs

**Name and Sequence (5' -> 3')**	**Length**	**# Reads**	**MDV/IRL Position**
mdv1-miR-M1: TGCTTGTTCACTGTGCGGCA^1^	20	304	136873
mdv1-miR-M2: GTTGTATTCTGCCCGGTAGTCCG^1^	23	191	134231
mdv1-miR-M2*: CGGACTGCCGCAGAATAGCTT^1^	21	16	134270
mdv1-miR-M3: ATGAAAATGTGAAACCTCTCCCGC^1^	24	13	134080
mdv1-miR-M4: TTAATGCTGTATCGGAACCCTTC^1^	23	206	134368
mdv1-miR-M4*: AATGGTTCTGACAGCATGACC^1^	21	6	134405
mdv1-miR-M5: TGTGTATCGTGGTCGTCTACTGT^1^	23	62	133647
mdv1-miR-M5*CGTATGCGATCACATTGACACG	22	12	133609
mdv1-miR-M6: GAGATCCCTGCGAAATGACAGT^1^	22	87	142370
mdv1-miR-M6*: TGTTGTTCCGTAGTGTTCTCG	21	39	142335
mdv1-miR-M7: TCGAGATCTCTACGAGATTACAG^1^	23	15	142547
mdv1-miR-M8: GTGACCTCTACGGAACAATAGT^1^	22	50	142258
mdv1-miR-M8* TATTGTTCTGTGGTTGGTTTCG	23	11	142216
mdv1-miR-M9: TGTTGATCCGTAGATAGGCGATGGC	25	5	96961
mdv1-miR-M10: GCGTTGTCTCGTAGAGGTCCAG^1^	22	4	142627
mdv1-miR-M10*: TCGAAATCTCTACGAGATAACAGTT	25	2	142669
mdv1-miR-M11: TTGCATAATACGGAGGGTTCTG	22	3	133925
mdv1-miR-M12: TGCTACAGTCGTGAGCAGATCAA	23	10	136581

### Potential novel microRNAs

About 10% of the reads matched the chicken genome but not other known small RNAs and were considered candidates for novel microRNAs. The presence of hairpin structures containing these reads was evaluated using RNAfold [10], and those present in hairpins were further filtered according to established criteria [11]. First, the candidate microRNA is entirely within the arm of the hairpin that has the lowest free energy among all sliding windows containing the candidate microRNA. Second, at least sixteen of 22 nucleotides of the candidate microRNA must match the other arm of the hairpin. Third, the hairpin should not contain any large (> 5 nt) internal loops or bulges. Matches to repeats or regions of low complexity were eliminated. Additional File 2 lists 63 candidate novel microRNAs passing these criteria. Uracil, which is preferentially found in the first position of known chicken microRNAs, is also first in 48% of the candidate novels. None contain a seed sequence that is identical to already established microRNA families. Three of the candidates (ID #26/27, 38/39 and 50/51) are found in the same stem loop, making it likely that they are mature and '*' strands of premicroRNAs. One (ID #10) is clustered 96 nt upstream of gga-miR-7-2, and one (ID#31) is immediately upstream of gga-let-7a-2.

Curiously, one of the potential novels (#ID14) is found within the highly evolutionarily conserved coding region of DCGR8 (DiGeorge syndrome critical region gene 8), which interacts with Drosha in the processing of pri-microRNAs [12].

**Figure 2:**
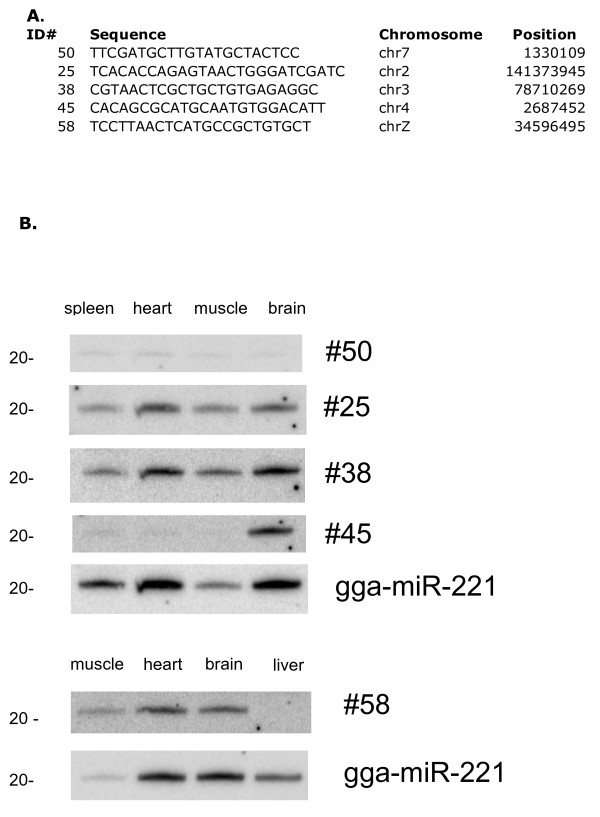
**Sequence and expression of novel chicken microRNAs**. A. Sequence and chromosomal location of selected novel microRNAs (location is based on May 2006 build). B. Northern blot analysis of individual microRNAs shows relative expression in different tissues. Blots were hybridized to gga-miR-221 to verify presence of microRNAs in each lane.

The expression of several of these candidate microRNAs was evaluated by northern blot analysis of different tissues (Figure 2). All hybridize to species the size of mature microRNAs. Some of the novel microRNAs are expressed ubiquitously (ID#26, 39, 51), while others show more selective expression (ID#46,61). These microRNAs show no sequence similarity to any known microRNAs, with the exception of #46, which is similar, but not identical to dre-miR-730 (21/22) and gga-miR-460 (19/22). The microRNAs analyzed by Northern blots were selected based on presence of star strand in sequencing data, presence in a cluster, or some level of sequence conservation with other species. Other candidate microRNAs in the list have not been evaluated.

### Expression of known host microRNAs in MDV-induced tumors

**Figure 3:**
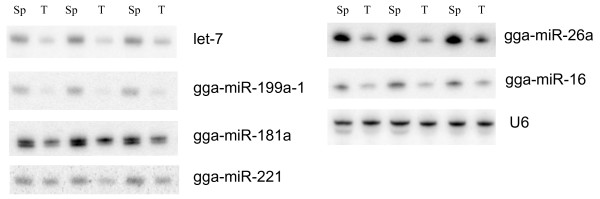
**Expression of chicken microRNAs in MDV-induced splenic tumors and normal spleens**. Small RNA from three individual MDV-induced splenic tumors (T) and normal spleen (Sp) were analyzed by Northern blot analysis and hybridization to probes antisense to indicated microRNAs.

There is a large and growing literature on microRNA expression in tumors, and both up- and down-regulation have been observed, with microRNA expression patterns reflecting the developmental history and lineage of neoplasms. We compared expression in MDV-induced tumors versus normal spleen tissue for selected host microRNAs that were either differentially expressed based on the deep sequencing or that were interesting based on the literature. Figure 3 shows that the expression of gga-miR-let 7, 199a-1, 26a, 181a, and -16 were all expressed at lower levels in tumors, compared to normal spleens, using either gga-miR-221 or U6 as a loading control; gga-miR-221 is slightly lower in tumors.

## Discussion

Our deep sequencing approach to microRNA discovery in the chicken confirms the expression of 112 known microRNAs and identifies a pool of 63 candidate novel microRNAs. The majority of the known chicken microRNAs have been identified by sequence comparison with microRNAs from other species [13], and the expression of many has been confirmed by analysis of EST data and *in situ *hybridization [14,15]. Cloning from small RNA libraries also validates the expression of microRNAs. A recent study of chicken microRNA cloning [16] used conventional technology to confirm expression of 25 of the known chicken microRNAs and identified one possible novel microRNA. Our study adds to the confirmation of expression of predicted microRNAs, and greatly expands the list of potential novel microRNAs in the chicken.

A large majority (86%) of the reads from chicken CEF small RNA libraries matched known microRNAs. Similar numbers were obtained in deep sequencing of human brain [6], where 80% of the reads matched known human microRNAs. Thus, it could be argued that we are approaching saturation of microRNA discovery. However, it is possible that only highly and ubiquitously expressed microRNAs have been found, and less abundant or tissue-specific microRNAs may still be revealed by deep sequencing of different tissues. This is clearly the case in plants, where a large set of small RNAs have been discovered by deep sequencing [3]. In an analysis of the human 'colorectal microRNAome', SAGE was used as a deep sequencing approach, and matches to 200 microRNAs in miRbase were found, as well as 100 previously unrecognized microRNA* strands and 133 candidate novels [17]. MPSS analysis of mouse embryo small RNA discovered over 60 potential novel microRNAs, some of which are rodent specific [11]. We have identified a pool of 63 candidate novel chicken microRNAs and have confirmed expression of several candidates by northern blot analysis. Some are expressed in a tissue-specific manner, while others are more ubiquitously expressed. Further analysis of these and other candidates with respect to temporal and tissue expression will be a first step to understanding function. Overall, the deep sequencing approach to microRNA discovery suggests that a significant number of novel microRNAs remain to be discovered and characterized.

In addition to identifying novel chicken microRNAs, deep sequencing of MDV infected CEF has revealed previously uncharacterized MDV-encoded microRNAs, bringing the total of MDV microRNAs to 18. Other herpesviruses encode microRNAs, and these are thought to play a role in immune evasion, apoptosis and cell cycle control [18,19]. MDV causes a well- characterized, virally induced T cell lymphoma of chickens and represents an excellent model system for analyzing the function of viral microRNAs in the pathogenesis of cancer. Many recent studies implicate microRNAs as either tumor suppressors or oncogenes [20], and host encoded microRNAs can act in *cis *(on viral target genes) or *trans *(on host encoded genes) to affect phenotypic changes [21]. Moreover, virally infected cells are stressed, and it has been proposed that microRNAs play an important role in the stress response [22]. A comparison of the reads of MDV-infected CEF versus uninfected CEF indicates that the majority of the microRNAs are expressed at similar levels. However, CEF are used to propagate virus, and viral infection occurs in a very small percentage of cells, thus making it difficult to observe changes when analyzing whole cultures. In addition, CEF are not the *in vivo *target of the virus, and we might expect a more critical layer of regulation in T cells. A small set of microRNAs appeared to be differentially expressed in infected vs. uninfected cells, but this was not confirmed by northern blot analysis. This lack of concordance between the two techniques is not uncommon when expression levels are very low, or when cross-hybridization with similar species can confound the results. Additionally, in our system, MDV infects only a small percentage of the cells and infections vary considerably from culture to culture. This biological noise could also hamper the ability to reproduce differences noted in the sequencing data set.

The two most frequently sequenced microRNAs were gga-miR-222 and -221, which also share sequence identity in the seed region. These are located within a 3000 nt region of Chromosome 1, where there are two copies of gga-miR-222 followed by one copy of gga-miR-221. In human, miR-221 and -222 are coordinately expressed from a single primary transcript [23]. We see about 1.7-fold higher abundance of ggg-miR-222 compared to gga-miR-221, consistent with their sharing a transcript that is highly expressed in CEF. Computational analysis has predicted that p27Kip1 protein, a key inhibitory regulator of the cell cycle [24], is a potential target for this cluster. Down regulation of p27Kip1 by miR-221/222 promotes growth and proliferation of cancer cells, and could play a similar role in dividing CEF [24]. miR-125b and 21 were also abundant in our libraries. miR-125b is critical for the proliferation of some human cell lines [25], and mir-21 is thought to function as an oncogene by decreasing apoptosis [26]. The high levels in rapidly dividing CEF could play a permissive role in the cell cycle in CEF.

Our northern analysis of MDV-induced tumors shows several host microRNAs that were noticeably less abundant in MDV-induced tumor tissue compared to normal spleen, consistent with previous reports of a general down-regulation of microRNAs in tumors [27]. Among those down regulated, let7 was particularly interesting. The let7 microRNA is known to down-regulate Hmga2 [28], which is a small, non-histone, chromatin-associated protein that is believed to influence chromatin remodeling [29]. Hmga2 is expressed robustly in undifferentiated proliferating cells, and its expression during embryogenesis and in a variety of benign and malignant tumors has been characterized [30]. Down-regulation of let7 should lead to increased expression of Hmga-2, and such a scenario would be consistent with the cell proliferation that characterizes tumors. miR-16 is considered a tumor suppressor [31], which acts by targeting BCL-2, and repressed expression is consistent with tumorigenesis. MiR-181s were down-regulated in gliobastoma compared to normal brain controls [32], and miR-199a was down-regulated in hepatocelluar carcinoma [33]. Thus, it is likely that in MDV-induced tumors, as in other tumors, many microRNAs act collectively to facilitate cellular transformation and proliferation. More information on the perturbations of host microRNAs will come from a deep sequencing analysis of microRNAs in tumors.

## Conclusion

Understanding the biological function of microRNAs first requires identification of all microRNAs within a genome. Here we have described the application of deep sequencing technology for the identification of many candidate novel chicken microRNAs from a single tissue source. The application of this technology to other tissues will no doubt lead to the identification of other novel microRNAs, which will improve the annotation of the chicken genome and further our understanding of this important class of regulatory molecules.

## Methods

### Cloning, sequencing and analysis of chicken microRNAs

Secondary CEF, prepared by routine techniques, were infected with the RB1B strain of MDV as described previously [9]. Protocols developed previously in the Green lab were used to construct the small RNA libraries [3]. cDNA inserts were amplified by PCR, and amplicons were sequenced by 454 Life Sciences [34]. Sequence data were filtered for adapter sequences, clustered (allowing a 4-base overhang or mismatch at either end), and insert sequence was analyzed by comparing to the chicken and MDV genomes, chicken ncRNA (Ensembl 12/06) and to the microRNA database [2] using Perl string matching. The remaining sequences were analyzed using RNAfold [10] to identify the loop structure of minimum free energy containing the microRNAs. This list was further curated to eliminate highly repetitive sequences [35].

### Northern blot analysis of microRNAs

RNA was fractionated using PEG or through use of the FlashPage system (Ambion) as described previously [9]. The low molecular weight fractions were electrophoresed on a 15% denaturing polyacrylamide gel, electroblotted to charged nylon, and hybridized to ^32^P-labeled antisense primers complementary to the microRNAs. Hybridization to an antisense primer for gga-miR-221 or U6 was used as a loading control. A 10-bp DNA ladder was used to approximate size.

## Authors' contributions

JB and RM designed and coordinated the study and wrote the manuscript; MO performed the analysis of the sequencing data; AA, EB and GI prepared samples for sequencing and for northern blots; CL prepared the libraries; PG and BM assisted in design, library construction and sequence analysis.

## Supplementary Material

Additional file 1 Expression of known microRNAs in CEF. Click here for file

Additional file 2 Candidate novel microRNAs. Click here for file
